# Cystatin C Secretion in Blood Derivatives and Cellular Models of Idiopathic Parkinson's Disease

**DOI:** 10.1155/padi/5149071

**Published:** 2025-01-24

**Authors:** Kathleen Mommaerts, Anna S. Monzel, Alise Zagare, Sarah L. Nickels, Nico J. Diederich, Laura Longhino, William Mathieson, Paul M. A. Antony, Fay Betsou, Jens C. Schwamborn

**Affiliations:** ^1^Integrated Biobank of Luxembourg, Luxembourg Institute of Health, Dudelange, Luxembourg; ^2^Luxembourg Centre for Systems Biomedicine, University of Luxembourg, Esch-sur-Alzette, Luxembourg; ^3^Department of Neurosciences, Centre Hospitalier de Luxembourg, Luxembourg City, Luxembourg; ^4^Centre de Ressources Biologiques de l'Institut Pasteur, Institut Pasteur, Paris, France

## Abstract

Parkinson's disease is an age-related progressive neurodegenerative disorder. A large majority of Parkinson's disease patients have an unknown etiology, which is classified as idiopathic Parkinson's disease. Generating disease models directly from idiopathic Parkinson's disease patients may improve the understanding of the disease pathology. Both neuroprotective and neurodegenerative roles have been suggested for cystatin C in neurodegenerative disease. In Parkinson's disease, investigations assessing cystatin C levels in different types of biospecimens such as blood, cerebrospinal fluid, and in vitro models have had conflicting results. We present a study assessing cystatin C levels in different biospecimen types originating from the same subjects. Using a sandwich ELISA, we compared cystatin C concentration in blood derivatives (plasma and serum) and culture media of derived models (stem cells, neuroepithelial stem cells, and midbrain organoids) of three idiopathic Parkinson's disease and three age-matched healthy control subjects with the same *CST3* genotype. Genotyping analyses confirmed that all subjects were homozygous AA. The identity of the derived models was confirmed using immunohistochemical staining. Secreted cystatin C concentration was measured in each biospecimen tested. No statistically significant differences in cystatin C concentrations were found between the subjects in any of the biospecimen types. As a personalized marker, a higher cystatin C concentration was shown for some individual patients in the culture medium of midbrain organoids, suggesting a potential involvement in Parkinson's disease physiopathology. This proof of concept demonstrates that cystatin C is secreted by various idiopathic Parkinson's disease cell models, including midbrain organoids, which in turn suggests that cystatin C secretion by midbrain organoids might be pertinent in neurodegenerative disease research.

## 1. Introduction

Parkinson's disease (PD) is an age-related progressive neurodegenerative disorder. The pathological hallmarks are the selective loss of dopaminergic neurons from the substantia nigra and the presence of protein aggregates (i.e., Lewy bodies) in the residual neurons in which α-synuclein is the primary structural component. Several genes have been associated with familial PD, and their investigation has given essential insights into molecular pathways engaged in the disease [1]. However, a large majority of PD patients have an unknown etiology, which is classified as idiopathic PD (iPD). Generating disease models directly from iPD patients may improve the understanding of the disease pathology.

It is possible that cystatin C plays an influential role in neurodegenerative disorders. Cystatin C expression levels have been shown to increase in neurodegenerative disorders and brain injury, but the exact role it plays in neurons is unclear, with both neurotoxic and neuroprotective functions being assigned to it in the literature [2–7]. Cystatin C is a cysteine protease inhibitor produced by most cells, which can be found in all body fluids and tissues [8, 9]. The mature enzyme is a low molecular mass protein (13.3 kDa), containing 120 amino acids. Cystatin C is encoded by the *CST3* gene located on chromosome 20 [8]. Previous studies report a major allele haplotype of G/A/G (allele A) and a minor allele haplotype of C/C/A (allele B) of the *CST3* gene [10, 11]. This polymorphism can affect cystatin C expression and secretion levels [12]. Cystatin C concentration is age-dependent, and age is also a risk factor for PD [13, 14]. Cystatin C is more abundant in cerebrospinal fluid than in blood, suggesting a functional role in the central nervous system [9].

We hypothesized that cystatin C levels might have diagnostic value in neurodegenerative disease when evaluated in the culture media of patient-derived cellular models, independently of their concentration in the blood, because in the latter, concentrations are dependent on the glomerular filtration rate and cells types that are not relevant to neurodegenerative diseases [15]. We tested this hypothesis by comparing cystatin C concentrations in the plasma and serum of three iPD patients and controls as well as in the culture media from cellular models (induced pluripotent stem cells (iPSCs), neuroepithelial stem cells (NESCs), and midbrain organoids (MOs)) generated from the same subjects.

## 2. Materials and Methods

### 2.1. Ethical Approval and Clinical Tests

Informed written consent was obtained from all study participants. The study was approved by the Luxembourg Research Ethics Committee (CNER, decision 201107/03). Six patients were selected: three with iPD and three healthy controls. iPD diagnosis was accordant with the London Brain Bank criteria. All patients maintained their usual medication, and both patients and controls underwent the following clinical evaluations for PD symptoms and cognitive impairment: the Hoehn and Yahr scale, the motor segment of the Unified Parkinson Disease Rating Scale (UPDRSIII), and the University of Pennsylvania Smell Identification Test (UPSIT). Significant disorders comorbidities, in particular renal and thyroid dysfunctions, were not reported. The demographics and clinical data of the patients are presented in Table 1.

### 2.2. Cystatin C Genotyping

Patients and controls were age-matched as well as matched with respect to genotype (homozygous AA). The *CST3* genotype was established by Sanger sequencing of three single-nucleotide polymorphisms of the *CST3* gene using an Applied Biosystems 3730xl DNA analyzer and BioEdit *v* 7.2.5 software: −82 G/C (rs5030707), +4 A/C (rs73318135), and +148 G/A (rs1064039).

### 2.3. Blood Derivatives Preparation

Serum was collected in 10 mL CAT Vacutainer tubes, left to clot, and then centrifuged at 2000*g* for 10 min. Plasma was collected in 10 mL K_2_EDTA Vacutainer tubes and then centrifuged at 2000*g* for 20 min. Both serum and plasma were stored at −80°C until analyses.

### 2.4. Generation of iPSCs

Human iPSCs were reprogrammed from fibroblasts that had been isolated and cultured following a published protocol [16]. Fibroblasts were mRNA-reprogrammed using either the Simplicon RNA Reprogramming kit (Millipore) (iPD patient S3) or with enhanced Sox/Oct transcription factors, as previously described [17]. iPS cells used in this study were cultured on plates coated with Matrigel (Corning, cat no. 354277) with iPSC medium consisting of Essential 8 Medium (Thermo Fischer, cat no. A1517001) supplemented with 1% Pen/Strep (Gibco, cat no. 15140). iPSC medium was exchanged daily. Expansion was performed by mechanical passaging with 0.5 mM EDTA (UltraPure 0.5M EDTA (ThermoFisher Scientific, cat no. 15575020) diluted in 1x D-PBS). iPSC characterization results are in Supporting Figure S1. 24-h incubated culture media were collected from 4 days postseeded iPSCs.

### 2.5. Generation of NESCs

Human NESCs were generated from iPSCs by small molecule patterning [18, 19]. NESCs were cultured on Matrigel-coated plates (Corning) in N2B27 media freshly supplemented with 3 μM CHIR-99021 (Axon Medchem), 0.75 μM purmorphamine (Enzo Life Science), and 150 μM ascorbic acid (Sigma) (referred to as N2B27 maintenance media) as previously described [19, 20]. N2B27 maintenance media consist of Neurobasal (Invitrogen) and DMEM-F12 (Invitrogen) (1:1), supplemented with 1:200 N2 supplement, 1:100 B27 supplement lacking Vitamin A (Invitrogen), 1% L-glutamine (Gibco), and 1% PenStrep. For maintenance, cells were typically split 1:10 every 7 days using Accutase (Stem Cell Technologies). NESC characterization results are in Supporting Figure S2. 48-h incubated culture media were collected from NESCs 4 days post-seeding.

### 2.6. Generation of MOs

Human MOs were generated from a single-cell suspension of NESCs following Accutase treatment as previously described [19–21]. NESCs were plated into ultralow attachment 96-well plates (Corning) at 9000 cells/well. Until Day 6, cells were cultured under maintenance conditions. On Day 6, the newly formed spheroids were transferred into 24-well plates (Celltreat) to clean and prepare them for embedding. On Day 8, the spheroids were embedded in Geltrex Basement Membrane Matrix (ThermoFisher Scientific) and then transferred to fresh 24-well plates at one spheroid/well. On Day 10 of culture, differentiation was initiated with N2B27 media, supplemented with 10 ng/mL hBDNF, 10 ng/mL hGDNF, 500 μM dbcAMP, 1 ng/mL TGF-β3 (all PeproTech), and 200 μM ascorbic acid (Sigma). 1 μM purmorphamine (Enzo Life Science) was additionally added to the media until Day 16 of culture, and from Day 14, the organoids were placed on an orbital shaker (IKA) rotating at 80 rpm. Media were changed every 3-4 days, and at Day 33, the MOs and 72-h-incubated culture media were collected for analyses. Cell seeding and incubation times had been consistent for all subjects and cellular models during culture. Culture media were stored at −80°C before analyses.

**Figure 1:**
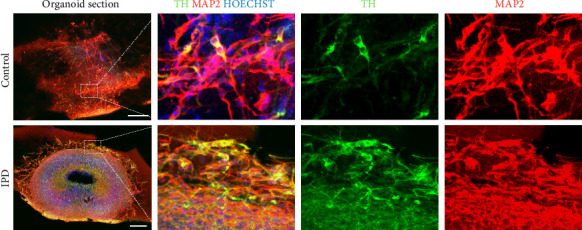
Characterization of the midbrains organoids by immunohistochemical staining for neuronal (MAP2) and dopaminergic (TH) markers at Day 30 of differentiation. Section imaging was performed using high-content automated microscopy. Scale bars represent 200 μm.

MOs were characterized as previously described [19–21]. Organoids were fixed overnight in 4% paraformaldehyde, embedded in agarose, and then sectioned at a thickness of 80 μm using a VT1000 S vibratome (Leica). The sections were permeabilized for 30 min in 0.5% Triton X-100 in PBS and then blocked for 2 h in blocking buffer (2.5% goat serum, 2.5% BSA, 0.1% Triton X-100, and 0.1% sodium azide). All incubations were performed at RT unless otherwise stated. The sections were then incubated for 48 h at 4°C in blocking buffer containing the primary antibodies rabbit antityrosine hydroxylase (TH) (1:1000, Abcam) and mouse anti–microtubule-associated protein 2 (MAP2) (1:1000, Abcam). Detection was then performed by incubating the sections in blocking buffer containing multiplexed Alexa-Fluor-conjugated secondary antibodies (Invitrogen). Nuclei were counterstained using Hoechst (1:1000, Invitrogen). Images were acquired on an Operetta automated confocal microscope (Perkin Elmer). Single images were stitched and processed using MATLAB (MathWorks). MO characterization results are presented in Figure 1.

### 2.7. Cystatin C Quantification

A sandwich ELISA was used to quantify cystatin C concentrations [22]. Plasma, serum, and the culture media required a 200x, 100x, and 2x dilution in sample diluent, respectively. An aliquot of fresh culture media was also assayed to verify it contained no cystatin C prior to incubation with the cellular models (iPSCs, NESCs, and MOs). For iPSCs and NESCs, the cystatin C concentration (ng/mL) was normalized to the number of viable cells at the point the culture media were collected.

### 2.8. Data Analysis and Availability Statement

All analyses were performed using the Mann–Whitney *U* test, using RStudio *v* 2022.07.1 with significance level *p* < 0.05. Data are available from the first author upon request.

## 3. Results

For cystatin C quantification analyses, genotyping confirmed that all subjects were homozygous AA. iPSC and NESC characterization results are presented as Supporting Figures S1 and S2, respectively. The spatial organization and identity of MOs were confirmed using immunohistochemical staining (Figure 1), using the neuronal marker MAP2 and the dopaminergic neuronal marker TH.

**Figure 2:**
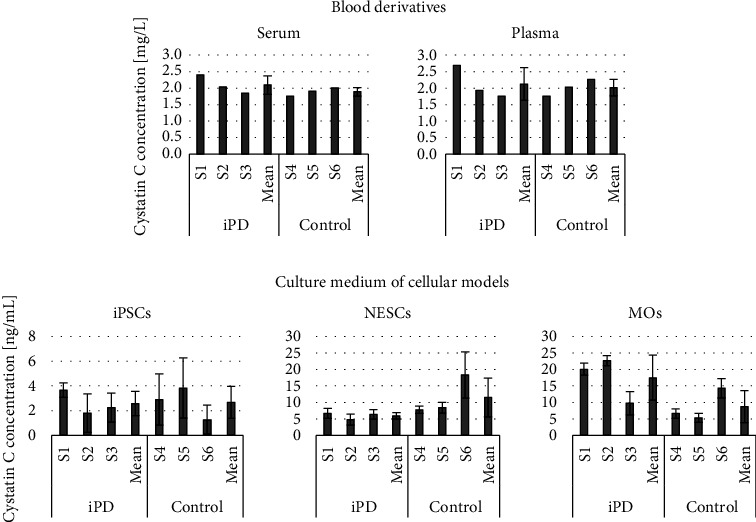
Cystatin C concentration in idiopathic Parkinson's disease (iPD) and controls. Error bars are standard deviation; iPSCs, induced pluripotent stem cells; MOs, midbrain organoids; NESCs, neuroepithelial stem cells.

No statistically significant differences were found between the iPD and control subjects (Figure 2).

In blood derivatives, the concentration (mean ± standard deviation (SD) (mg/L)) for iPD and controls, respectively, were 2.10 ± 0.28 and 1.89 ± 0.13 in serum and 2.13 ± 0.50 and 2.02 ± 0.25 in plasma. In the culture media, the concentration (mean ± SD (ng/mL)) for iPD and controls, respectively, were 2.57 ± 0.97 and 2.67 ± 1.30 for iPSCs, 5.93 ± 1.01 and 11.51 ± 5.91 for NESCs, and 17.49 ± 6.86 and 8.74 ± 4.86 for MOs. The normalized concentration (mean ± SD ((ng/mL)/cells × 10^6^) for iPD and controls, respectively, was 1.57 ± 0.59 and 1.85 ± 0.44 for iPSCs and 40.86 ± 35.88 and 66.81 ± 24.93 for NESCs.

## 4. Discussion

We used a sandwich ELISA to quantify the cystatin C concentration in serum and plasma from iPD patients and controls, as well as in the culture media of in vitro models (iPSCs, NESCs, and MOs) derived from the same individuals. Given that polymorphism in the *CST3* gene can affect cystatin C expression and secretion from cells, genotyping of a larger cohort of subjects was performed so the genotype (homozygous AA) of patients and controls could be matched. From the initial nine subjects genotyped, six were homozygous AA and three were heterozygous AB. This was concordant with the published genotype frequency, where the AA genotype is the most common (75.7%), followed by the AB genotype (22.5%) and the BB genotype (1.8%) [23].

Cystatin C concentrations in plasma correlated highly with those in serum (*r* = 0.93, *p*=0.008), which is consistent with published data [24]. In addition, the observed concentrations in plasma were comparable with the expected range of 1.1 ± 0.42 mg/L [9]. We demonstrated that cystatin C is secreted by all tested cellular models, including the MOs. To the best of our knowledge, this has not been previously reported. Taking into account the different incubation times, when the cystatin C concentration in cell culture media was normalized to the number of viable cells, NESCs had higher concentrations than iPSCs. This is consistent with a previous study that demonstrated cystatin C supports neuronal stem cell proliferation and differentiation [2].

We found no statistically significant differences in cystatin C concentration between iPD patients and controls in the blood derivatives or in the in vitro models. In blood derivatives, increased expression of cystatin C of patients suffering with neurodegenerative disorders has previously been reported, which appears contradictory to our findings [25–28]. However, our group of iPD patients did not have the cognitive impairment or motor dysfunction that is associated with increased cystatin C concentration in the plasma and serum of patients suffering with PD. In the in vitro MO model, when performing the Mann–Whitney *U* test comparing cystatin C concentrations in culture media of MOs derived from iPD and controls, no statistically significant difference was found (*p*=0.2). However, when looking at the patients individually, we note that two of them (S1 and S2) were responsible for driving the higher mean cystatin C concentration compared to that of the controls, and the third patient (S3) had a similar cystatin C concentration as the controls. These differences in cystatin C concentration between the three PD patients may be a consequence of differences in disease duration (S1: 10 years, S2: 5 years, and S3: 2 years (Table 1)). However, the iPD patients with the lower disease progression (S2 and S3) did not have correspondingly lower scores on the UPSIT and UPDRS scales (denoting less severe symptoms). It is therefore possible that the cystatin C concentration in MOs might be useful in the stratification of PD subtypes.

Of the three in vitro models tested, the midbrain cellular model is more representative of the in vivo human midbrain than iPSCs or NESCs. The in vitro presence of different cell types (as in the MOs) is essential to more accurately reflect the in vivo human midbrain, because the secretion of cystatin C by one cell type might impact another cell type. The use of regionally patterned NESCs as a starting population for MOs has the advantage that already-patterned cells differentiate efficiently into the desired structures, which reduces the variability of the generated MOs [29]. In particular, MOs at 30 days of differentiation have shown to recapitulate cardinal features of PD, such as loss of dopaminergic neurons [30] and protein aggregation [31].

Cystatin C was selected as a protein of interest as changes in its expression, and secretion levels have been found in the brains of patients with diverse neurodegenerative conditions and in human and animal models of neurodegeneration. In Alzheimer's disease (AD), cystatin C has been shown to have a neuroprotective action by inducing autophagy, inhibiting cysteine proteases and by inhibiting amyloid-β aggregation [32]. Furthermore, the *CST3* BB genotype is associated with increased risk of developing AD. A neuroprotective action of cystatin C has also been demonstrated in the amyotrophic lateral sclerosis mouse model [33, 34] and in the progressive myoclonic epilepsy type 1 mouse model [35]. In PD, a study has demonstrated that the elevated expression of cystatin C prolonged the survival of dopaminergic neurons in the 6-hydroxydopamine (6-OHDA) rat model [36]. Another study showed the neuroprotective action of exogenously applied cystatin C in human neuronal cultures exposed to cytotoxic challenges [37, 38]. The authors showed that the neuroprotective action does not involve cathepsin B inhibition but the induction of fully functional autophagy via the mTOR pathway [38]. Another study, using in vivo and in vitro animal models of PD, found that exogenous cystatin C promotes neuronal survival through autophagic clearance of α-synuclein aggregates [39]. Taken together, these papers show that cystatin C has demonstrably a role in AD and potentially in other neurodegenerative diseases such as PD. Our hypothesis is that cystatin C does not restrict itself to protease inhibition but involves the induction of fully functional autophagy.

A limitation of this study is the small sample size, which requires validation in a larger cohort. In addition, only one male patient is included in the study, and PD is known to be more prevalent in males than females [40]. However, it is generally accepted that the predominance of PD in the male gender is caused by different hormone levels [41]. Given that the cell culture model we used was independent of gender-based hormonal differences (no hormones were added to the culture medium), we think it justified not to differentiate between male and female. The major strengths of our study were the inclusion of subjects with comparable clinical parameters and the utilization of different biospecimen types that were derived from the same PD patients and controls, which facilitated an evaluation of the most appropriate biospecimen type for cystatin C quantification in PD.

## 5. Conclusions

This proof of concept demonstrates that cystatin C is secreted by various iPD cell models, including MOs, which in turn suggests that cystatin C secretion by MOs might be pertinent in neurodegenerative disease research.

## Figures and Tables

**Table 1:** Clinical data.

	Diagnostic	Gender	Age at sampling (years)	*CST3* genotype	Disease duration (years)	Hoehn–Yahr score	UPDRS motor score	Schwab England (%)	UPSIT score
S1	iPD	Female	67	AA	10	1	6	100	21
S2	iPD	Female	67	AA	5	1	13	80	22
S3	iPD	Male	71	AA	2	1	8	100	10
S4	Control	Female	65	AA	NA	0	0	100	33
S5	Control	Female	63	AA	NA	0	2	100	32
S6	Control	Female	68	AA	NA	0	0	100	25

Abbreviations: NA = Not Applicable, UPDRS = Unified Parkinson's Disease Rating Scale, UPSIT = University of Pennsylvania Smell Identification Test.

## Data Availability

Data are available from the first author upon request.

## Supporting Information

Supporting Figure S1: iPSC quality and integrity were verified by confirming normal diploid karyotypes and positive alkaline phosphatase staining, typical expression of stem cell markers (SSEA4, NANOG, TRA-1-60, OCT4, TRA-1-81, and SOX2) by immunocytochemistry, and robust expression levels of stem cell marker genes (OCT4, SOX2, and NANOG) by qRT-PCR. Self-renewing capability was verified using immunofluorescence, with primary antibodies targeting NANOG (Millipore, AB5731, 1:100), SSEA4 (Millipore, MAB4304, 1:25), OCT4 (Abcam, ab19857, 1:600), TRA-1-60 (Millipore, MAB4360, 1:25), SOX2 (Abcam, ab97959, 1:200), and TRA-1-81 (Millipore, MAB4381, 1:25). Secondary antibodies were anti-mouse Alexa Fluor 488 and anti-rabbit Alexa Fluor 568 (Invitrogen, A21202 and A10042, respectively, 1:1000). A nuclei counterstain Hoechst 33342 (ThermoFisher Scientific, H3570, 1:10 000) was included. A representative image set for sample S1 can be seen in Figure S1.

Supporting Figure S2: NESC quality was verified by confirming typical expression of stem cell and neurectoderm markers (NESTIN, SOX2, PAX6, KI67, and SOX1) by immunocytochemistry. NESCs were stained for NESTIN (BD Bioscience, 1:200), SOX2 (R&D systems, 1:200), PAX6 (Biolegend, 1:300), KI67 (GenScript, 1:200), and SOX1 (R&D systems, 1:200). Hoechst 33342 (ThermoFisher Scientific, H3570, 1:10 000) was used for nuclei staining. A representative image set for sample S1 can be seen in Figure S2.
