# Supplementary material for: Cystatin C Secretion in Blood Derivatives and Cellular Models of Idiopathic Parkinson's Disease
Source: Parkinsons Dis. 2025 Jan 24;2025:5149071. doi: 10.1155/padi/5149071 (PMC11824396; doi:10.1155/padi/5149071)

**Cystatin C secretion in blood derivatives and cellular models of idiopathic Parkinson’s disease**

**SUPPLEMENTARY MATERIALS**

**Supplementary Figure S1**


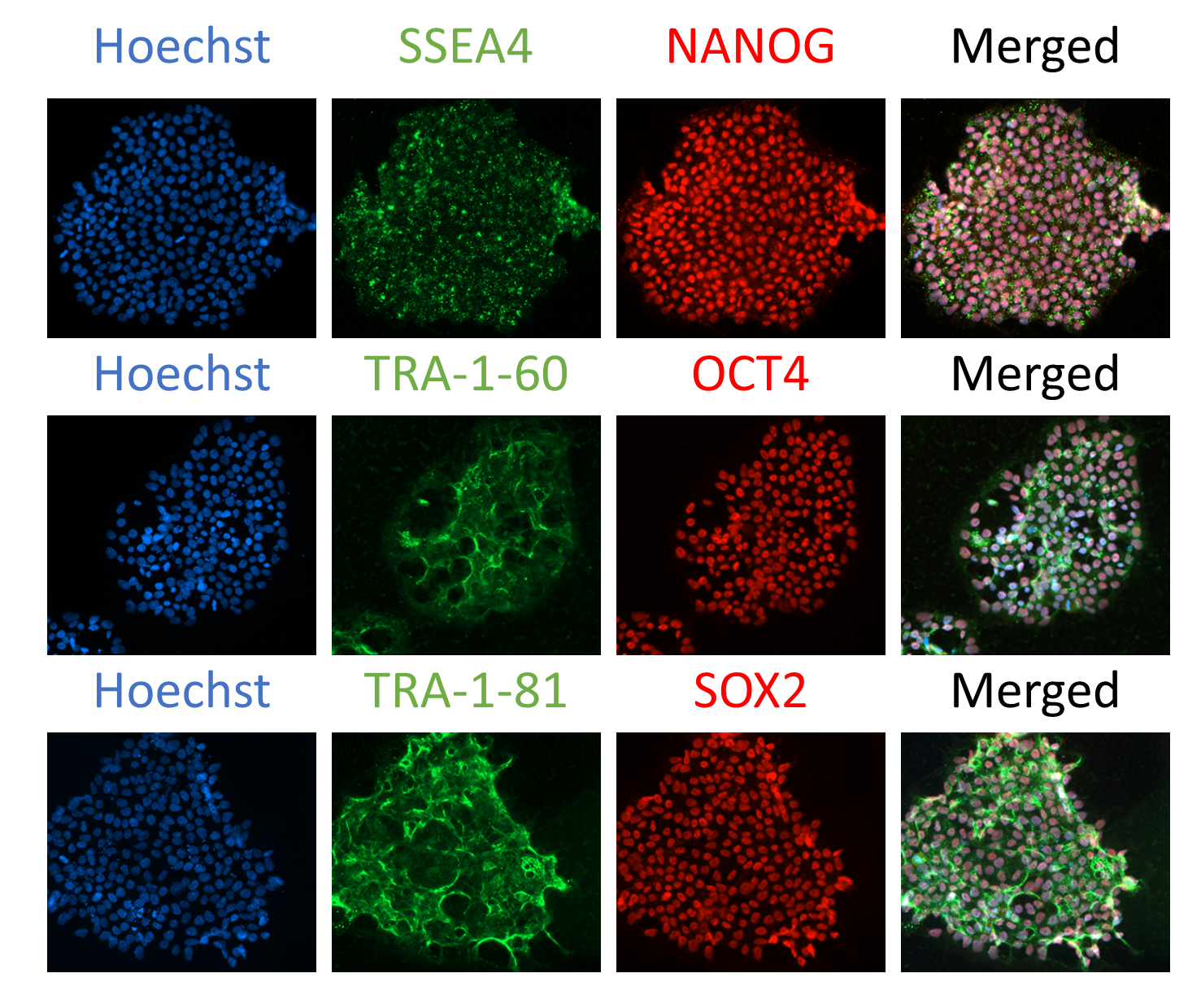


**Supplementary Figure S2**
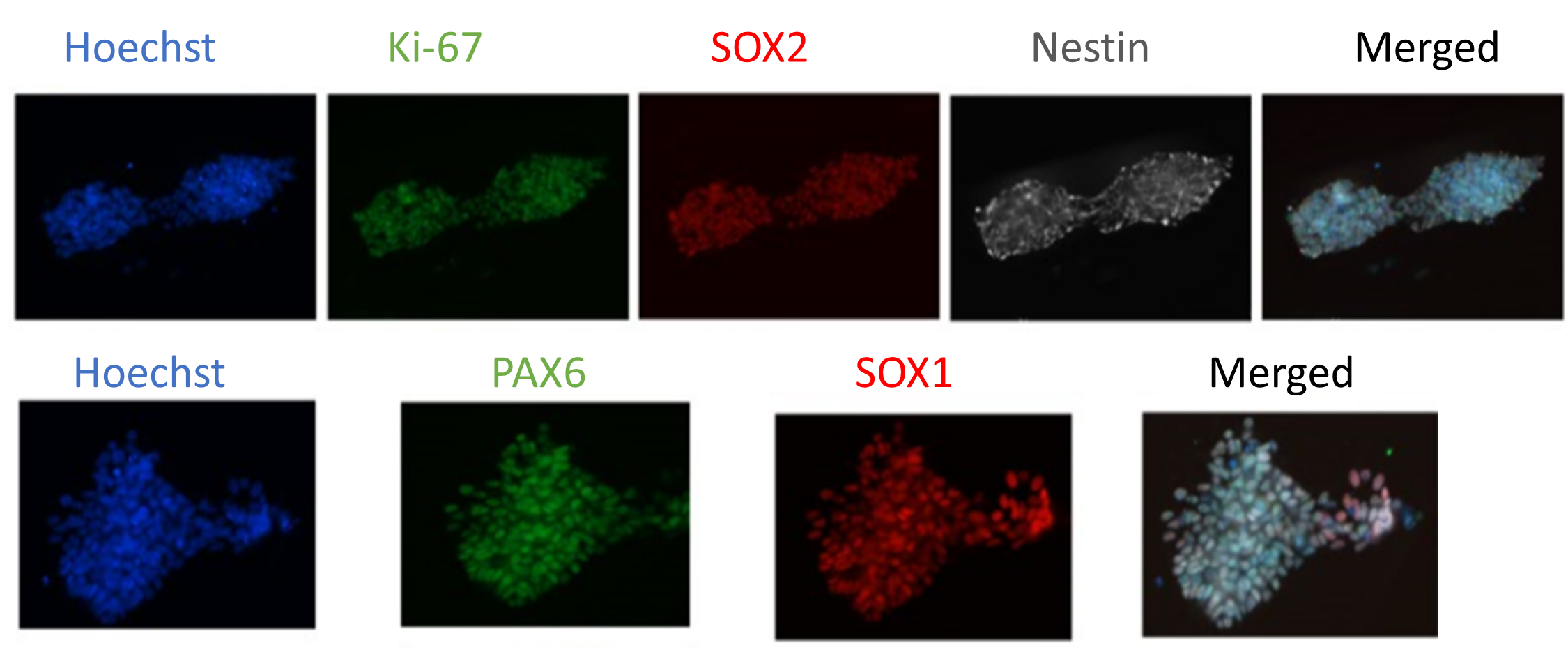

Supplement: Supporting Information — Additional supporting information can be found online in the Supporting Information section. [file 5149071.f1.docx]
